# Effects of *Cynodon dactylon* on Stress-Induced Infertility in Male Rats

**DOI:** 10.4103/0975-1483.76416

**Published:** 2011

**Authors:** VR Chidrawar, HR Chitme, KN Patel, NJ Patel, VR Racharla, NC Dhoraji, KR Vadalia

**Affiliations:** *Department of Pharmacology, Shree H. N. Shukla Institute of Pharmaceutical Education and Research, Gujarat, India*; 1 *Department of Pharmacology, Oman Medical College, Azaiba-Muscat, Sultanate of Oman*; 2 *Department of Pharmacognosy, Arihant School of Pharmacy and Bio-Research Institute, Gandhinagar, Gujarat, India*; 3 *Department of Pharmacology, Saraswati Institute of Pharmaceutical Sciences At: Dhanap, Dist. Gandhinagar, Gujarat, India*; 4 *Drug Regulatory Affairs, Emcure Pharmaceutical Pvt. Ltd., Pune, Maharashtra, India*; 5 *Department of Pharmacology, H.S.K. College of Pharmacy, Bagalkot, Karnataka, India*; 6 *Department of Pharmaceutical Chemistry, Shree H. N. Shukla Institute of Pharmaceutical Education and Research, Rajkot, Gujarat, India*

**Keywords:** *Cynodon dactylon*, infertility, sperm count, stress, testosterone

## Abstract

*Cynodon dactylon* (Family: Poaceae) is known to be a tackler in Indian mythology and is offered to Lord Ganesha. It is found everywhere, even on waste land, road side, dry places, and spreads vigorously on cultivated ground. This study was carried out with an objective to test if the constituents of this plant are useful in coping stress-induced sexual In this study, we considered immobilization stress to induce male infertility and the effect of *C. dactylon* in restoration of the dysfunction was evaluated by considering sexual behavioral observations, sexual performance, fructose content of the seminal vesicles, epididymal sperm concentration and histopathological examinations as parameters. Treatment of rats under stress with methanolic extract of *C. dactylon* has shown a promising effect in overcoming stress-induced sexual dysfunction, sexual performance, fructose content, sperm concentration and its effect on accessory sexual organs and body weight. We conclude that active constituents of *C. dactylon* present in methanolic extract have a potent aphrodisiac and male fertility activity.

## INTRODUCTION

Stress is a major factor in rising health care costs; excessive amounts of stress hormones in brain tissue cause the nerve cells, or neurons, in parts of the hippocampus to wither and eventually die. Stress affects sexual desire in both men and women and can cause impotence in men. It appears to worsen the symptoms of premenstrual syndrome in women, cause amenorrhea or cessation of menstrual periods and affects fertility. Stress during pregnancy is associated with a 50% higher risk of miscarriage.[1]

In men, both physical and psychological stress may interfere with the reproductive capacity adversely, activation of the hypothalamic-pituitary-adrenal axis by stressors is a presumable mechanism for the inhibition of male reproductive functions through a depression in the hypothalamic-pituitary-testicular axis.[2,3] Acute and chronic immobilization attained in studies of experimental stress can decrease testosterone secretion in males, and as testosterone plays an important role in the sexual performance and sexual act, stress seems to be a predominant factor in the male infertility.[3] Several reports have suggested a stress related decline in semen quality, sperm concentration, morphology and percentage of motility. This leads to a close correlation between the sperm concentration and fertility potential of males.[4]

In the present study, we propose to systematically evaluate the effect of *Cynodon dactylon* on stress-induced changes in sexual behavior. Recently, it has been reported to have hypotensive property and slowing of blood flow in mesenteric capillaries of rats. This effect was supported by involvement of nitric oxide (NO) and free radical scavenging activity.[5]

*C. dactylon*, most commonly known as Dhruva, is an elegant perennial grass, growing throughout India. Traditionally, fresh expressed juice of the grass is useful in hemuturesis, vomiting and as application in catarrhal opthalmia, and also can be applied to cuts and wounds as it checks bleeding, and in chronic diarrhea and dysentery. Decoctions of root are used in vesical calculus and secondary syphilis, stoppage of bleeding from piles, and irritation of urinary organs.[6] An alkaloid isolated from the plant slows down the flow of blood in mesenteric capillaries, has hypoglycemic effect, causes reduced bleeding and clotting time, hypotension, has antioxidant activityand antiviral activity against the vaccinia virus.[7] However, till date, no sufficient systemic and scientific studies have been carried out on the pharmacological and phytocompositions of *C. dactylon*, except on its *in vitro* antioxidant activity, antimicrobial activity and wound healing property.[5,8,9]

## MATERIALS AND METHODS

### Materials

The materials used for the study were Remi research centrifuge (R-24), Soxhlet extractor, Research microscope (Metzer) and Handy cam (Sony), WBC diluting pipettes, and Neubaur’s counting chamber of hemocytometer.

Testosterone injections (25 mg/ml) (Aquaviron) were purchased from Nicholas (Mumbai, India), sildenafil citrate from Cipla Pharmaceuticals, (Mumbai, India), progesterone injections (250 mg/ml), from Almet Corporation, (Mumbai, India), and estradiol valerate injections (10 mg/ml), (Progynon Depote, German Remedies, Mumbai, India) were purchased from the local market. All the chemicals and solvents used in this study were of AR grade.

### Methods

In the present study, the aerial part of *C. dactylon* was collected from the Ayurvedic College, Bagalkot. The aerial parts were authenticated by Taxonomist, B.V.V.S. Ayurvedic College, Bagalkot, Karnataka, and confirmed by Botanist, Basaveshwar Science College, Bagalkot, Karnataka. After authentication, all the aerial parts were cleaned and washed with 2% KMNO _4_ prepared in distilled water and then dried at room temperature, until they were free from moisture. Finally, the aerial parts of plant were subjected to get a coarse powder and then passed through sieve no. 44 to get uniform powder. The sieved powder was stored in airtight, high-density polyethylene containers until extraction.

#### Plant preparation

The powder was subjected to hot, continuous, successive extraction (soxhlet), a 12-hour cycle with benzene and 90% methanol (5055°C). Finally, the drug was macerated for 24 hours with water. After the effective extraction, the solvent was distilled off and the excess solvent was completely removed by using rotary flash evaporator to get a semisolid mass. Percentage yield was calculated with respect to weight of the dried plant material. The obtained extracts were subjected to pharmacological screening for determining their role in stress-induced sexual behavior in adult male rats. The percentage yields of benzene, methanol and water extracts were 3.31, 8.96 and 6.32%, respectively.[10]

#### Acute oral toxicity

Healthy Swiss albino mice of either sex, weighing 15–20 g, starved overnight, were divided into three groups (*n* = 6) and fed with increasing doses (1, 2 and 5 g/kg) of each extract and the toxicity was evaluated as per the Guidelines for Non-clinical Toxicity Investigation of Herbal Medicine given by Ministry of Health and Family Welfare, Government of India.[11] All the drug extracts administered orally in doses of up to 5 g/kg did not produce any evident toxicity. Mortality in mice was observed up to 14 days after administration (not shown).

#### Animal selection

Healthy, sexually experienced male Wistar albino male rats weighing 250–300 g and female rats weighing 180200 g were used in this study. The animals were kept in well-ventilated animal house conditions with free access to pelleted food and tap water throughout the experiment. All the pharmacological experimental protocols were approved by the Institutional Animals Ethics Committee (HSKCP/IAEC.Clear/2006-2007, dated 27 Dec 2007), H.S.K. College of Pharmacy, Bagalkot, Karnataka.

#### Aphrodisiac activity

The animals were divided into five groups of six animals each, in which three groups were received methanolic extract in doses of 100, 200 and 500 mg/kg, respectively. First group served as normal which received vehicle (2 ml/kg) of 2% dimethyl sulfoxide (DMSO) p.o. The second group served as standard and received sildenafil citrate 0.7 mg/kg (i.p.); all the treatments were given for 10 consecutive days.[12,13]

For the evaluation of aphrodisiac activity, these rats were individually placed in cages 3 hours following the administration and were given 10 min adaptation period. A separate female rat was taken for each male. Every female that had been brought into ouestrus (oectradiol benzoate 12 μg in olive oil injected subcutaneously 56 hours prior to pairing plus progesterone 0.5 mg in olive oil injected subcutaneously 8 hours prior to pairing) was placed in the cage before pairing.[14]

The following parameters of sexual behavior were monitored 15 min after pairing, under dim light and video recording was done using Sony handycam. Latency of first mount, number of mounts, latency of first intromission, inter intromission interval, number of intromissions, latency of first ejaculation, latency of second ejaculation, average ejaculation latency and number of ejaculations was recorded.

#### Stress-induced sexual behavior in male rats

A total of 60 adult sexually experienced male Wistar albino rats weighing 250–300 g were taken and randomly divided into 10 groups. Group I served as normal and received only 2 ml/kg of the vehicle, i.e., 2% DMSO. Group II served as control and received 2 ml/kg of vehicle (2% DMSO) and was subjected to stress by immobilization (IMB). Group III served as standard and received testosterone (0.05 mg per rat, i.m.) and was subjected to stress by IMBgroups IV and V received methanolic extracts 100 and 200 mg/kg, respectively, and IMB stress; groups VI, VII and VIII received benzene extracts 100, 200 and 500 mg/kg, respectively, and IMB stress; groups IX and X received water extracts 100 and 200 mg/kg, respectively, and IMB stress. All extracts were given by oral gavages.[15]

#### Induction of IMB stress

The animals were subjected to IMB stress by Plexiglas cylinder (5 cm diameter and 16 cm large) for 6 h day during light period starting from 8 a.m. each day for 30 consecutive days. Water and food were withdrawn during the stress period.[16]

#### Observation of sexual behavior

Stress-induced change in the sexual behavior and effect of extracts on these behaviors was studied by following the same aphrodisiacal protocol and parameters.[15]

#### Organ to body weight ratio

Body weight of each animal was measured before the IMB stress and drug treatment was started. Percentage change in the body weight was calculated by weighing the animals just before sacrificing them. After sacrificing each animal, accessory sexual organs; testis, vas deferens, prostate glands, seminal vesicles, epididymis and adrenal glands were isolated and weighed in a wet condition to measure organ to body weight ratio.[17]

#### Assessment of fructose content and sperm count

Right hemiprostate and seminal vesicle with the adherent prostate gland were removed and stored at –20°C until the fructose content in seminal vesicle was determined by the method of Linder and Mann.[18] Sperm was collected from the left cauda epididymis and the concentration of spermatozoa was estimated in the sperm suspension stored in the cauda epididymis, according to the method described by Kempinas and Lamano-Carvalho.[19]

#### Histology

Two left testis of each group were excised and rinsed in 0.9% saline, and were blotted dry of saline and excess blood. They were fixed in 12% formalin for 24 hours. The tissues, after fixation, were washed in water to remove the excess fixative. Washed tissues were then dehydrated through a graded series of ethyl alcohol, cleared with xylene and embedded in paraffin wax. Sections of 3 μm thickness were cut with a microtome blade, and mounted on clean glass slides. The sections were routinely stained with hematoxylin and eosin. The stained slides were observed (400 ×) in research microscope and photographed.

### Statistical analysis

Data collected in the study were expressed as the mean ± SEM and statistical analysis was carried out using student’s *t*-test. *P* value less than 0.05 was considered to be statistically significant.

## RESULTS

### Effect of methanolic extract of *Cynodon dactylon* (CDME) on aphrodisiac potential in male Wistar rats

CDME was selected for testing aphrodisiac potential in normal male rats prior to fertility activity, based on its high percent yield (6.32%)

As per the earlier studies, sildenafil citrate (7 mg/kg) used in our study significantly (*P* < 0.01) reduced latency time of first mount, first intromission, and first ejaculation, when compared to DMSO treated normal male rats. Sildenafil citrate also significantly increased the total number of mounts (31.66 ± 2.97) and inter intromission interval (252.5 ± 14.24) (*P* < 0.001), indicating potent male aphrodisiac property [Table 1]. Dose-dependent, significant effects were observed on treatment of male rats for 10 days with CDME (100, 200, and 500 mg/kg), with respect to the total number of mounts and inter intromission interval, Latency of first mount was significantly (*P* < 0.01) increased (42.26 ± 8.9) by the highest dose of methanolic extract. High dose of *C. dactylon* methanolic extract significantly increased the sexual performance in male rats by increasing ejaculation latency when compared to group I rats. Latency of first intromission was significantly decreased by 500 mg/kg of CDME in comparison to control male rats.

**Table 1:** Effect of CDME on aphrodisiac potential of male rats

Treatment	Latency of first mount (sec)	No. of mounts	Latency of first intromission (sec)	Inter intromission interval (sec)	No. of intromissions	Latency of first ejaculation (sec)	Latency of second ejaculation (sec)	Ave. ejaculation latency (sec)	No of ejaculations
Vehicle DMSO 2% (2 ml/kg, p.o.)	289.16 ± 44.23	6.5 ± 1.28	545 ± 19.61	126.66 ± 8.816	2	251.66 ± 2855	130 ± 5.77	177.5 ± 24	1.83
Sildenafil citrate (7 mg/kg, i.p.)	16.5 ± 3.63**	31.66 ± 2.97***	242.5 ± 22.44	252.5 ± 14.24***	2	159.16 ± 18.63*	151.66 ± 11.07	155.41 ± 9.31	2
CDME (100 mg/kg, p.o.)	240 ± 0.97	11.33 ± 1.33*	530 ± 30.5	145 ± 12.84	1.33	270 ± 20.48	150 ± 17.31	175 ± 14.31	1.5
CDME (200 mg/kg, p.o.)	195.83 ± 33.04	16.5 ± 0.43*	339.16 ± 38.79	249.16 ± 27.33	2	165.83 ± 25.04	658.33 ± 30.45	467.083 ± 61	2
CDME (500 mg/kg, p.o.)	42.26 ± 8.9**	19.66 ± 1.78**	187.83 ± 10.58***	290 ± 2.88***	2	103.5 ± 8.3**	542.5 ± 19.81***	323 ± 8.78**	2

*n* = 6. Effect of 2% DMSO, sildenafil citrate (7 mg/kg, i.p.) and *C. dactylon* methanolic extract (100, 200 and 500 mg/kg; p.o.) on aphrodisiac potential was studied in male Wistar rats. All the drug treatments were given for 10 conjugative. Rats were individually placed in cages 3 hours following the administration and were given 10 min adaptation period. A separate female rat was taken for each male. Every female that had been brought into ouestrus was placed in the cage before pairing. Behavior of rats was recorded for 15 min each. All the data collected are presented as mean ± SEM and analyzed by student’s t test

*P* < 0.05 was considered significant

* *P* < 0.05

** *P* < 0.001

*** *P* < 0.0001

### Effect of *Cynodon dactylon* on body organ weight in adult Wistar albino rats

Change in body weight is an important parameter considered in most of the studies to recommend health status of animals during the experiment.

Wistar albino rats, when exposed to restrainer stress, significantly (*P* < 0.05) lost there body weight in comparison to group I rats [Table 2]. Intramuscular treatment of restrained animals with 15 mg/kg testosterone restored the body weight lost compared to DMSO treated stressed rats. High dose of *C. dactylon* benzene extract (500 mg/kg) significantly (*P* < 0.01) caused a regular increase in the body weight to 11.56 ± 0.939, followed by small (100 mg/kg), and moderate doses (200 mg/ kg) of the extract and DMSO treated group. Also, 200 mg/kg of CDME significantly (*P* < 0.05) potentiated stress-induced fall in body weight from 18.54 ± 0.88 to 14.5 ± 0.746, no significant alteration in the body weight of stressed rats was seen when treated with *C. dactylon* benzene (CDBE) and water extracts 100 and 200 mg/kg (CDWE).

**Table 2:** Effect of *Cynodon dactylon* on stress modulated body and vital organ weights in adult male rats

Treatment	% Change in body weight	Wet weight of isolated accessory sexual organs to body weight ratio (mg/g)					
		Testis	Vas deferens	Seminal vesicles	Adrenal gland	Prostate gland	Epididymis
Vehicle DMSO 2%, normal rats	35.281 ± 6.304	8.074 ± 0.511	0.286 ± 0.031	2.115 ± 0.107	0.099 ± 0.006	1.419 ± 0.113	1.706 ± 0.096
Vehicle DMSO 2%, stressed rats	18.54 ± 0.882*	4.3 ± 0.471**	0.195 ± 0.007*	1.228 ± 0.153**	0.045 ± 0.004**	0.963 ± 0.024**	1.041 ± 0.061**
Testosterone (15 mg/kg, i.m.)	21.94 ± 1.033	7.702 ± 0.275**	0.258 ± 0.027**	2.268 ± 0.106	0.081 ± 0.005**	1.156 ± 0.116**	1.457 ± 0.180
CDBE (100 mg/kg, p.o.)	16.55 ± 2.525	4.728 ± 0.13	0.2 ± 0.009	2.771 ± 0.071***	0.371 ± 0.33	1.639 ± 0.093***	1.540 ± 0.045**
CDBE (200 mg/kg;, p.o.)	13.47 ± 1.398*	4.81 ± 0.218	0.205 ± 0.008	3.030 ± 0.119***	0.044 ± 0.006	1.177 ± 0.133	1.505 ± 0.044**
CDBE (500 mg/kg;, p.o.)	11.56 ± 0.939**	4.102 ± 0.092	0.194 ± 0.006	2.278 ± 0.317*	0.030 ± 0.001*	1.394 ± 0.025***	1.313 ± 0.016**
CDME (100 mg/kg, p.o.)	14.5 ± 0.746*	4.807 ± 0.76	0.2 ± 0.011	2.186 ± 0.141**	0.058 ± 0.005**	1.127 ± 0.060	1.599 ± 0.196*
CDME (200 mg/kg;, p.o.)	18.91 ± 1.551	6.874 ± 0.178	0.274 ± 0.019**	2.317 ± 0.124	0.062 ± 0.013	2.004 ± 0.193**	2.357 ± 0.151***
CDWE (100 mg/kg;, p.o.)	16.07 ± 0.88	4.233 ± 0.226	0.190 ± 0.024	2.277 ± 0.187**	0.059 ± 0.008	1.308 ± 0.047**	1.44 ± 0.061**
CDWE (200 mg/kg;, p.o.)	17.2	4.441 ± 0.086	0.272 ± 0.006***	2.736 ± 0.194**	0.044 ± 0.006	1.180 ± 0.146	1.389 ± 0.095*

Effects of DMSO (2%), testosterone (15 mg/kg, i.m.) and *C. dactylon* extracts on stress modulated body weight and organ to body weight ratio were studied in adult male rats on 30th day, after anesthesia with ether. Organs were isolated and weighed. The wet weight of organ to body weight was calculated and expressed as mean ± SEM. The results obtained were analyzed by student’s t test

*P* < 0.05 was considered significant

* *P* < 0.05

** *P* < 0.001

*** *P* < 0.0001

Oral administration of CDME (200 mg/kg) not only significantly increased the wet weight of vas deferens, seminal vesicles, prostate gland and epididymis, but also increased the wet weight of testis to body weight ratio from 6.874 ± 0.174, compared to the testis to body weight ratio of CDBE, CDWE and DMSO treated groups. However, restrained stress reduced significantly the wet weight of testis to body weight ratio, and this effect was reversed significantly by testosterone treatment (*P* < 0.01) in Wistar albino rats. In Table 2, it is seen that restrained stress induced significant decrease in wet weight of accessory sexual organs including testis, vas deferens, seminal vesicles, adrenal glands, prostate gland and epididymis, when compared to normal control rats. Testosterone treatment (15 mg/kg) not only significantly restored stress-induced reduction in testis, vas deferens, adrenal gland and prostate gland (*P* < 0.01), but also increased the wet weight of seminal vesicles and epididymis to body weight significantly.

CDBE and CDWE showed no significant change in testis wet weight to body weight rationwhen compared to restrain stressed and testosterone treated rats. CDME at both doses selected in this study significantly inhibited the stress-induced reduction in wet weight of isolated accessory sexual organs to body weight ratio.

### Effect of *Cynodon dactylon* on the amount of fructose content, sperm concentration and fertility of male rats

Wistar albino rats when exposed to restrainer stress showed significant reduction in sperm count (*P* < 0.05) and fructose content compared to normal vehicle and testosterone treated rats [Table 3]. Among the extracts, methanolic extract (200 mg/kg) showed significant restoration of sperm count (*P* < 0.05) and fructose content (*P* < 0.001) compared to benzene and water extracts, but the effect was lesser than the testosterone treated group.

**Table 3:** Effect of *Cynodon dactylon* on stress modulated sperm count and fructose content adult male rats

Treatment	Fructose content (mg/g) seminal vesicle	Sperm count (million/mm^3^) cauda epididymis
Vehicle DMSO 2%, normal rats	5.62 ± 0.21*	49.0 ± 3.8**
Vehicle DMSO 2% (2 ml/kg, p.o.) and IMB stress	3.23 ± 1.2	11.9 ± 2.5*
Std. testosterone (15 mg/kg, i.p.) and IMB stress	4.65 ± 0.36**	47 ± 3.2
CDBE (100 mg/kg, p.o.) and IMB stress	3.4 ± 1.23	14 ± 0.6**
CDBE (200 mg/kg, p.o.) and IMB stress	3.42 ± 1.36	15 ± 1.2
CDME (100 mg/kg, p.o.) and IMB stress	4.11 ± 0.26*	29 ± 1.5
CDME (200 mg/kg, p.o.) and IMB stress	4.52 ± 1.4**	43 ± 2.5*
CDWE (100 mg/kg, p.o.) and IMB stress	2.9 ± 0.47	17 ± 1.7**
CDWE (200 mg/kg, p.o.) and IMB stress	3.04 ± 0.21**	20 ± 0.2

All the data collected are presented as mean ± SEM and analyzed by student’s *t* test

*P* < 0.05 was considered significant

* *P* < 0.05

** *P* < 0.001

## DISCUSSION

In the present study, we demonstrated the aphrodisiac and fertility effects of *C. dactylon*, using stress-induced infertility models of male Wistar albino rats by considering sperm count, fructose content, health parameters, observation of sexual behavior, and histopathological examinations.

The present study on the effect of *C. dactylon* in male fertility activity of stress-induced infertile male rats was carried out based on our results of primary investigation conducted randomly with CDME. In the aphrodisiac activity evaluation, we obtained appreciable results with CDME at a dose of 500 mg/, similar to the effect shown by sildenafil citrate, suggesting its effectiveness in increasing male sexual performance, libido and possibly overcoming certain forms of male infertility like premature ejaculation.

**Figure 1:**
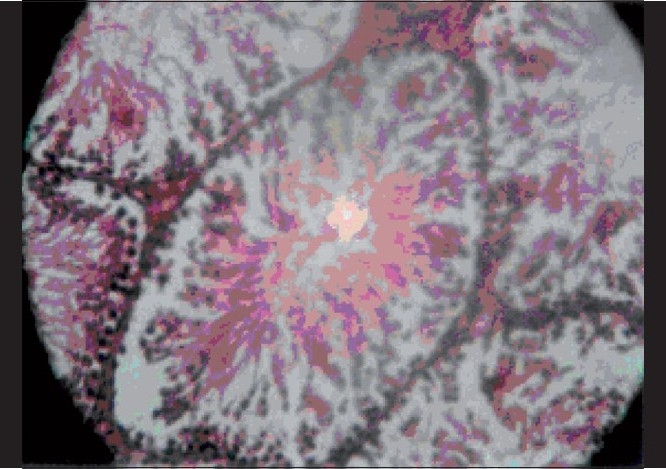
Effect of testosterone (15 mg/kg, i.m.) on histopathological changes in stressed rat. Basement membrane, spermatogonia, spermatocytes, spermatids and sertoli sells (H and E, ×400)

**Figure 2:**
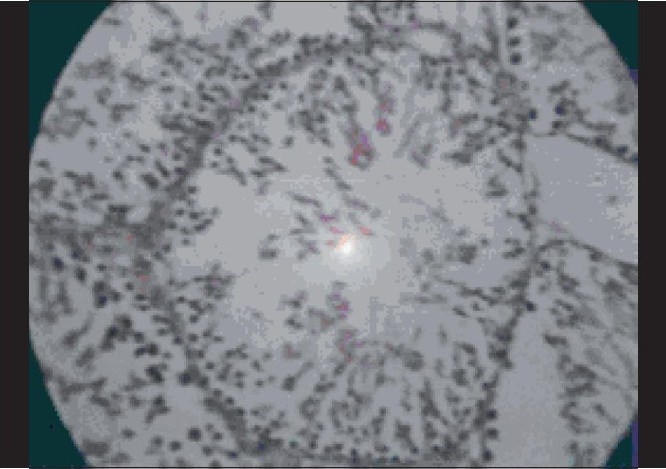
Effect of 2% DMSO on histopathological changes in stressed rat testis. Narrowing of seminiferous tubules, less spermatogenesis, low secondary spermatocytes and spermatids (H and E, ×400)

**Figure 3:**
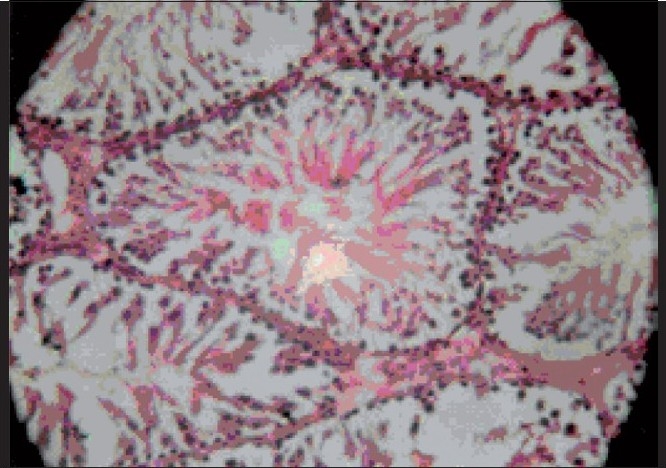
Effect of 2% DMSO on normal histology of rat testis. Normal arrangement of basement membrane, spermatogonia, spermatocytes, spermatids and sertoli cells (H and E, ×400)

**Figure 4:**
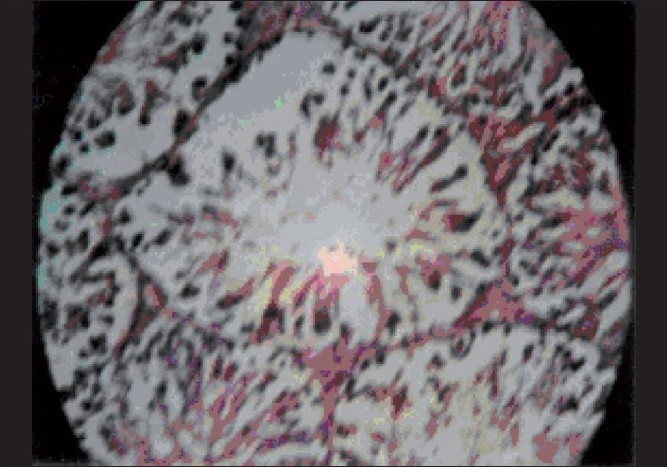
Effect of *Cynodon dactylon* benzene extract (100 mg/kg, p.o.) on histopathological changes in stressed rat testis. Enlarged seminiferous tubules, less spermatogenesis, low secondary spermatocytes and spermatids (H and E, ×400)

**Figure 5:**
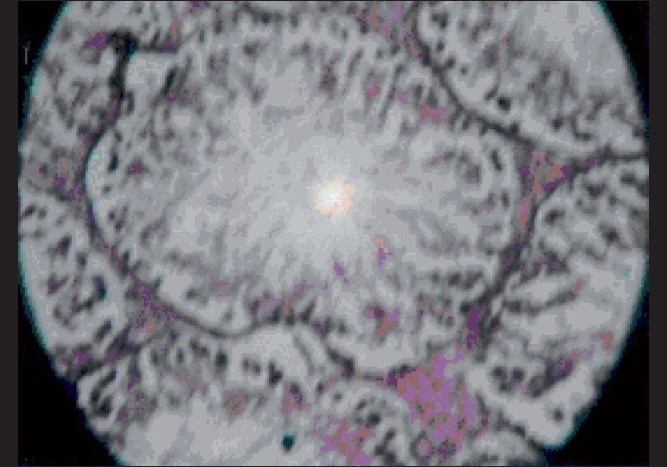
Effect of *Cynodon dactylon* benzene extract (200 mg/kg, p.o.) on histopathological changes in stressed rat testis. Enlargement of seminiferous tubules, thickening of basement membrane, less no. of spermatocytes, medium no. of sperm cells and sertoli cells (H and E, ×400)

**Figure 6:**
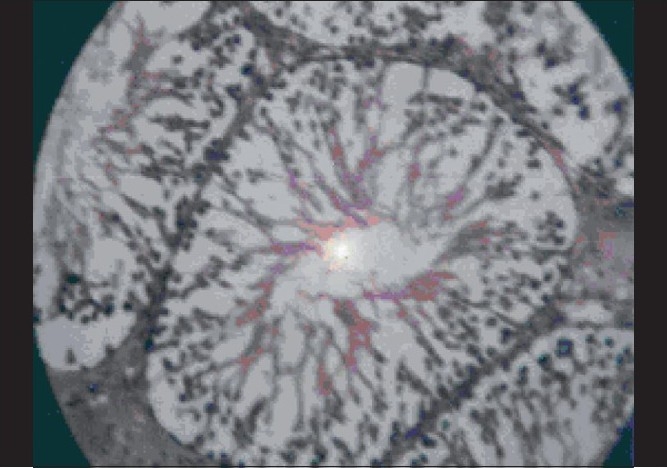
Effect of *Cynodon dactylon* benzene extract (500 mg/kg, p.o.) on histopathological changes in stressed rat testis. Normal no. of spermatocytes, spermatids, spermatogonium and sertoli cells (H and E, ×400)

**Figure 7:**
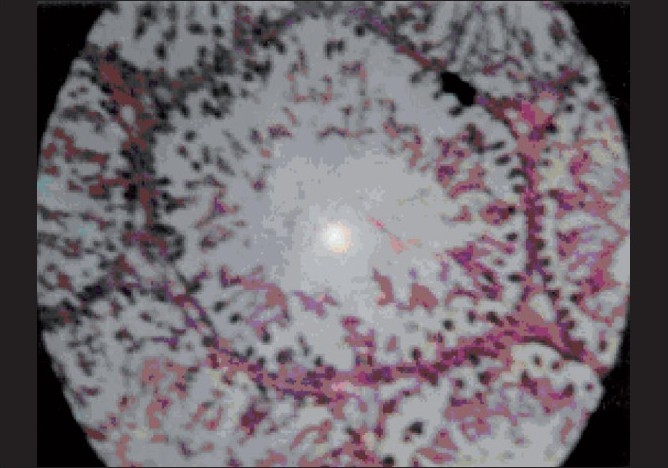
Effect of *Cynodon dactylon* aqueous extract (100 mg/kg, p.o.) on histopathological changes in stressed rat testis. Thin basement membrane, less no. of spermatogonia, sertoli cells and absence of sperm cells (H and E, ×400)

**Figure 8:**
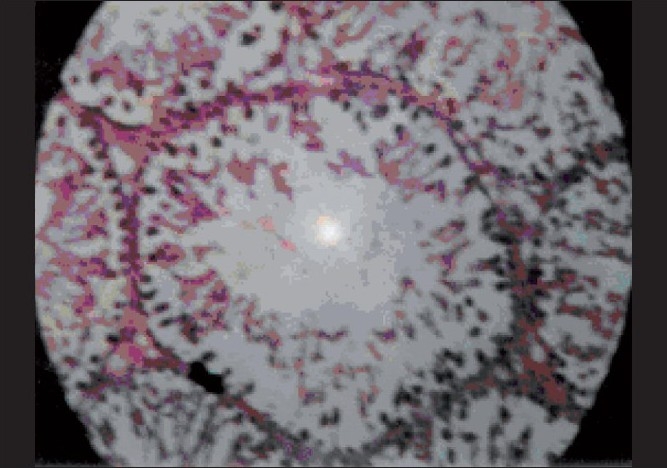
Effect of *Cynodon dactylon* aqueous extract (200 mg/kg, p.o.) on histopathological changes in stressed rat testis. Medium no. of sperm cells, spermatocytes, sertoli cells, spermatogonia and widening of seminiferous lumen (H and E, ×400)

**Figure 9:**
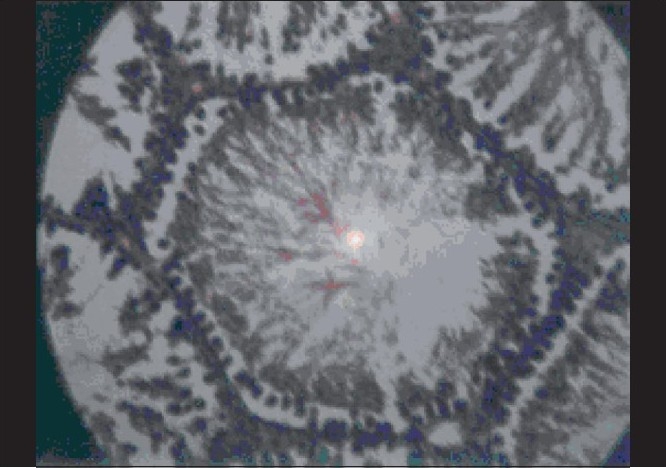
Effect of *Cynodon dactylon* aqueous extract (500 mg/kg, p.o.) on histopathological changes in stressed rat testis. Narrowing of seminiferous lumen, thickening of basement membrane and arrangement of sertoli cells toward the basement membrane(H and E, ×400)

**Figure 10:**
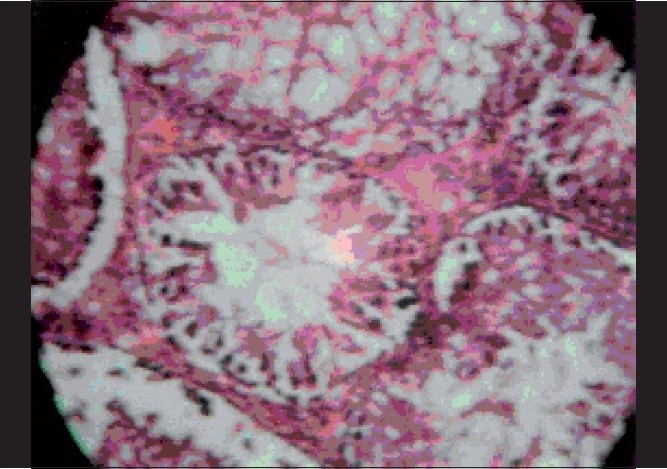
Effect of *Cynodon dactylon* methanolic extract (100 mg/kg, p.o.) on histopathological changes in stressed rat testis. Thickening of basement membrane, aggregation of spermatocytes, low no. of sperm cell and sertoli cells (H and E, ×400)

**Figure 11:**
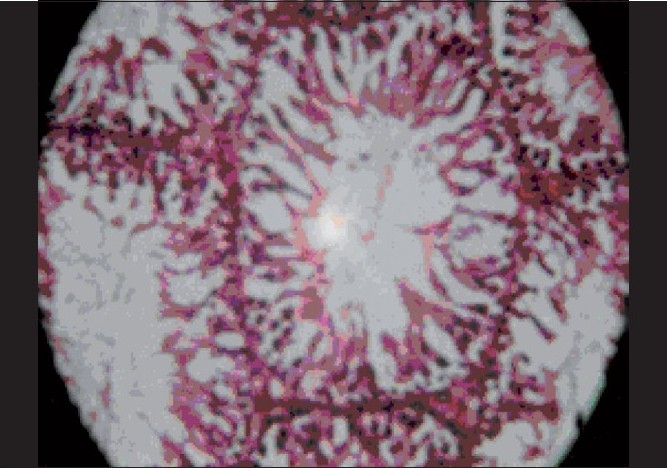
Effect of *Cynodon dactylon* methanolic extract (200 mg/kg, p.o.) on histopathological changes in stressed rat testis. Aggregation of spermatocytes, spermatids, spermatogonium and sertoli cells toward the basement membrane (H and E, ×400)

**Figure 12:**
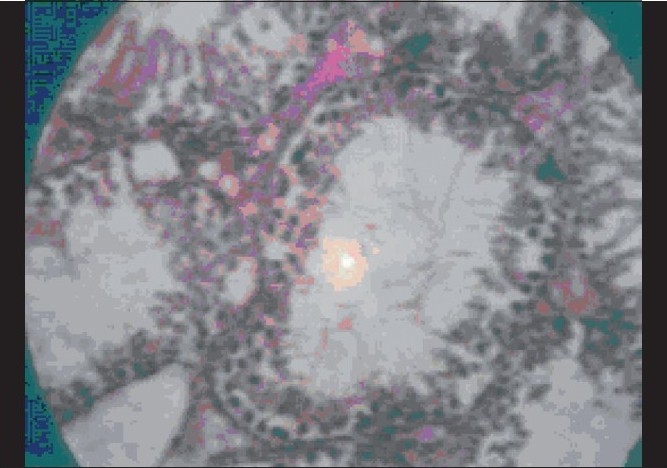
Effect of *Cynodon dactylon* methanolic extract (500 mg/kg, p.o.) on histopathological changes in stressed rat testis. Widening of seminiferous lumen, less no. of secondary spermatids, aggregation of interstitial cells toward the basement membrane, low no. of sperm cells and sertoli cells (H and E, ×400)

It has been well established that exposure to stressful conditions will lead to various physiopathological conditions including the effects on endocrine system. The affected endocrinal system activates hypothalamo-hypophyseal-gonadal system as well as hypothalamo-hypophyseal-adrenocortical along with neuroendocrine axis, which have a profound disruptive effect on male reproductive function and sexual act by the suppression of testosterone secretion, spermatogenesis and libido.[20] In our study, the group of the animals exposed to stress with a vehicle treatment showed similar results, i.e., stress decreased masculine sexual behavior and sperm count in rats.[21] It is well known that exposure to chronic stress induces lower circulating level of testosterone, prolactin, corticotrophin releasing hormone and also modulated adrenocorticotrophin (ACTH), beta-endorphin and glucocorticoids in hypothalamic-pituitary-gonadal axis function and impairs luteinizing-hormone-releasing-hormone (LH-RH) coordination.[22] In our study, results obtained from testosterone treated group [Table 4] of animals showed a significant decrease in latency of first mount, increase in total number of mounts, decrease in intromission interval, increase in the latency of first ejaculation and these results were complemented by increase in total sperm count, fructose content and histopathological examination [Figure 1]. Previous studies have indicated that fructose is a nutrient substance present in seminal plasma. This reducing sugar, if converted by the spermatozoa to lactic acid, provides an important source of energy for sperm cells. This was significantly (*P* < 0.001) increased and there was also significant (*P* < 0.05) rise in sperm count in methanolic extract treated group than benzene and aqueous extract treated groups [Table 2], but lesser than testosterone treated group.[23] These findings are also supported by histopathological examinations of testis, sexual behavior and organ to body weight ratio of accessory sexual organs. Histopathological examination on the last day in stressed rats showed a marked decrease in seminiferous tubular diameter bounded by loose intratubular connective tissue, reduction in spermatogenesis, increased interstitial spaces and morphological abnormality despite maintaining germinal epithelial architecture [Figure 2] compared with normal control group which represents normal architecture of the testis [Figure 3]. An increase in the total number of spermatogonium, spermatocytes and spermatids in testosterone treated groups [Figure 1] indicates that testosterone has significant ability in overcoming severe to chronic stress and restoring male sexual act and reproductive system. The postmortem study in group of animals treated with benzene and water extracts has shown significant infiltration of inflammatory cells, narrowing of vasculature and homeostasis in testis at various doses as showed in [Figures 4–6] and [Figures 7–9] respectively, specifically these disturbances are predominant in the high doses of benzene and aqueous extracts as shown in Figure 6 and Figure 12 respectively. Treatment of stressed animals with methanolic extract has shown a dose-dependent ability in restoring the architecture of testis, similar to the effect of testosterone [Figures 10–12]

**Table 4:** Effect of *Cynodon dactylon* on stress modulated sexual behavior in adult male rats

Treatment	Latency of first mount	No. of mounts	Latency of first intromission	Inter intromission interval	No. of intromissions	Latency of first ejaculation	Latency of second ejaculation	Ave. ejaculation latency	No of ejaculations
Normal sexual behavior	289.16 ± 44.23	6.5 ± 1.28	545 ±19.61	126.66 ± 8.816	2	251.66 ± 2855	130 ± 5.77	177.5 ± 24	1.83 ± 0.11
Vehicle DMSO 2% (2 ml/kg, p.o) and IMB stress	–	–	–	–	–	–	–	–	–
Std. testosterone (15 mg/kg mg/kg, i.p.) and IMB stress	351 ± 111.62*	11 ± 2.84*	5 ± 138.57*	95.25 ± 49.74	1.33 ± 0.33*	135.17 ± 33.9*	49.67 ± 22.66	92.33 ± 20.45**	1.33 ± 0.33*
CDBE (100 mg/kg, p.o.) and IMB stress	–	–	–	–	–	–	–	–	–
CDBE (200 mg/kg, p.o.) and IMB stress	–	–	–	–	–	–	–	–	–
CDME (100 mg/kg, p.o.) and IMB stress	7.66 ± 1.22	11 ± 0.8	447 ± 195.88	159 ± 3.86	2	440.33 ± 7.38	224.33 ± 5.72	335.66 ± 1.51	2
CDME (200 mg/kg, p.o.) and IMB stress	181.5 ± 12.16	19.5 ± 1.41*	259.33 ± 122.51*	70.83 ± 40.95	1.5 ± 0.284	455.5 ± 132.65	220	345 ± 132.65	1.5 ± 0.28
CDWE (100 mg/kg, p.o.) and IMB stress	238.66 ± 104.63	7.83 ± 3.63	420 ± 2.8	–	0.5 ± 0.22	245.5 ± 107.63	–	122.75 ± 53.82	0.5 ± 0.22
CDWE (200 mg/kg, p.o.) and IMB stress	127.83 ± 67.55	8.33 ± 3.06*	637 ± 120.49*	465	1.166 ± 0.392	121.33 ± 90.55	65 ± 31.98	126.5 ± 48.25*	1.167 ± 0.39

Effect of 2% DMSO, testosterone (15 mg/kg, i.p.) and *C. dactylon* extracts on stress modulated male sexual behavior was studied in Wistar rats. Animals were restrained for 6 hours/day till 30 days, along with treatment. Treatment was given 1 hour prior to their exposure to stress. After 30 days of stress, the animals were exposed to female rats, and the behavior of rats was recorded for 15 min each. All the data collected are presented as mean ± SEM and analyzed by student’s t test

*P* < 0.05 was considered significant

* *P* < 0.05

** *P* < 0.001

CDME significantly increased the mounting frequency and total number of mounts and decreased intromission interval and mount latency, as compared to controls and more than that of the standard drug Sildenafil citrate. However, CDBE and CDWE extracts failed to overcome stress-induced decline in sexual activity. This was an indication that the phytoconstituents of *C. dactylon* are present in the methanolic extract and also indicates that 200 mg/kg of methanolic extract improves the male sexual performance and may be useful in overcoming stress-induced premature ejaculation dysfunction.

Latency of first intromission is considered as an index of potency, libido and also a parameter of the rate of recovery from exhaustion after first series of mating.[24] This was significantly (*P* < 0.05) decreased with CDME and also with the testosterone treatment, but the effect with methanolic extract was more pronounced than with testosterone.

In stressed rats, reduction in the number of spermatids and spermatozoa present in the tissue accounts for reduced testicular weight.[25] Decreased weights of accessory sexual organs, prostate gland, seminal vesicles, vas deferens and epididymis indicate the atrophy of glandular tissue, diminished secretary ability and low level of testosterone, as these organs are androgen dependent.[26] Probably, this may be followed in our study as evidenced by significant increase in organ wet weight to body weight ratio of testis, vas deferens and prostate gland in testosterone treated groups.

The increase in wet weight of adrenal glands to body weight ratio by treatment with testosterone, methanolic extract, and benzene extract on rats under stress indicates the similarity in the effect of extracts and testosterone. Probably, these extracts produce their effects in the development of these accessory sexual organs through the hypothalamo-hypophyseal-adrenocortical system.

It has been well established that the estrogen is formed from testosterone by the sertoli cells, when they are stimulated by follicle stimulating hormone (FSH). The combined activity of testosterone and estrogen is probably essential for spermatogenesis and improving the male sexual function as estrogen has been widely used to abrogate male climacteric symptoms.[27] In phytochemical analysis, we found that there was presence of phytosterols and alkaloids in the methanolic extract; the benzene extract contained saponins and alkaloids; and the aqueous extract showed the presence of glycosides, carbohydrates, and saponins. From this study, we propose that phytosteroids undergo absorption and metabolism in the gastric tract and get converted into phytoestrogen metabolites. These phytoestrogen metabolites act in a way similar to estrogen derivatives and produce their effects through activation of estrogenic receptors. Estrogenic receptors may be present in testis, sertoli cells and leydig cells, thereby improving sexual performance and male reproductivity. Possibly, this may be one of the mechanisms in illustrating the effect of *C. dactylon* steroids present in methanolic and benzene extracts.

## CONCLUSION

Based on these results and earlier reports, we propose that *C. dactylon* may have important phytoconstituents which may be beneficial in promoting male sexual behavior in normal rats as well as stress-induced sexual dysfunction. Possibly, the active constituents may produce these effects probably mediated through hypothalamo-hypophyseal-adrenocortical axis, regulating abnormality of limbic system, estrogen modulating property of phytosteroids, and anti-stress activity of alkaloids and protective effect on accessory sexual organs.
